# Reaming-induced joint surface destruction in tibiotalocalcaneal nailing: a cadaveric comparison of straight and valgus-curved nails

**DOI:** 10.1186/s13018-026-07136-0

**Published:** 2026-07-28

**Authors:** Mahmut Kalem, Kamil Balaban, Malik Kısmet, Mehmet Yılmaz

**Affiliations:** 1 Department of Orthopedics and Traumatology, Faculty of Medicine, Ankara University, Ankara, Turkey; 2 Department of Orthopedics and Traumatology at the Ministry of Health Finike State Hospital, 07740 Finike, Antalya Turkey; 3 Department of Anatomy, Faculty of Medicine, Ankara University, Ankara, Turkey

**Keywords:** Subtalar joint, Hindfoot fusion, Intramedullary nail, Tibiotalocalcaneal, Cadaveric study

## Abstract

**Background:**

Tibiotalocalcaneal (TTC) nailing has become an increasingly preferred option for the treatment of complex ankle pathologies and fragility fractures, particularly in geriatric patients. Despite its growing clinical use, limited data are available regarding how different nail designs impact joint surface integrity during intraoperative preparation. This cadaveric study aimed to compare the articular surface destruction caused by straight and valgus-curved TTC nails.

**Methods:**

20 fresh-frozen cadaveric lower limbs (mean age 70.1 ± 4.1 years) were randomized to receive either a straight or a valgus-curved TTC nail (n = 10 per group). All procedures were performed by a single experienced orthopedic surgeon without formal joint debridement. After reaming, four joint surfaces—calcaneal posterior facet (CPF), talar posterior facet (TPF), talar dome (TD), and tibial plafond (TP)—were dissected and digitally analyzed using ImageJ software to measure total and reamed areas. Joint destruction (%) and articular reaming (%) were calculated and compared between groups.

**Results:**

Valgus-curved nails resulted in significantly greater joint destruction in CPF (12% vs. 6%, *p* = 0.018) and TPF (15% vs. 8%, *p* = 0.007) compared to straight nails. Articular reaming was also higher in the valgus-curved nails for both CPF (56% vs. 31%, *p* = 0.012) and TPF (79% vs. 43%, *p* = 0.004). No significant differences were found in TD or TP for either parameter (all *p* > 0.05).

**Conclusion:**

Reaming for valgus-curved TTC nails yielded greater destruction of subtalar joint surfaces than straight nails, but the amount remained below 15%. These findings emphasize the impact of nail design on reaming-induced articular surface destruction during intraoperative preparation and provide quantitative anatomic data on the extent of joint surface involvement.

**Supplementary Information:**

The online version contains supplementary material available at https://doi.org/10.1186/s13018-026-07136-0.

## Introduction

Retrograde intramedullary nails have gained significant renewed interest in recent years. This includes both established and emerging indications, largely driven by the technique’s success and minimally invasive profile [1, 2]. One such indication is for selected cases of complicated ankle fracture fixation, aiming to reduce wound complications and issues related to postoperative mobilization in elderly or low-demand patients with substantial medical comorbidities, including poorly controlled diabetes mellitus, renal failure, peripheral vascular disease, documented osteoporosis, and compromised soft tissue envelopes [3–6].

In elderly patients with low functional demands, tibiotalocalcaneal (TTC) nailing has increasingly been used as a primary fixation strategy for unstable ankle fractures, especially where traditional open reduction and internal fixation poses significant risks [4]. In this context, TTC nailing is often performed to provide mechanical stability rather than to achieve arthrodesis, without formal joint preparation [7, 8]. While this approach has demonstrated favorable outcomes and functional recovery, the long-term implications of retained articular cartilage—particularly in relation to implant stability, loosening, or failure—remain a subject of debate [9, 10].

While the literature has extensively examined the ideal entry point and the relationship of TTC nailing with neurovascular structures, the clinical rationale for choosing between straight and valgus-curved nails is also closely related to coronal plane alignment and safe calcaneal containment [11]. Because the calcaneal body lies lateral to the tibial canal axis, a strictly colinear straight nail trajectory may risk medial calcaneal cortical breach or require hindfoot medialization, whereas valgus-oriented trajectories may allow a more lateral calcaneal entry point and reduce the risk to medial plantar neurovascular structures [11–14]. However, only a few cadaveric studies have evaluated the extent of articular surface damage resulting from reaming [15–17]. Although these studies primarily evaluated the safety of specific nail designs and reaming-related risks, they showed varying levels of joint surface destruction, ranging from 4 to 20% at the tibiotalar and subtalar joints [18, 19]. Nonetheless, most cadaveric research has focused on a single nail type or surgical technique, and there remains a lack of comparative analyses evaluating articular damage caused by different nail designs, such as straight versus valgus-curved nails.

This cadaveric study investigated the effect of two commonly used TTC nail designs—straight and valgus-curved—on the articular surfaces of the tibiotalar and subtalar joints, with the aim of quantitatively comparing reaming-induced cartilage removal in a setting that simulates surgical preparation without formal joint debridement**,** as frequently encountered in TTC nailing for geriatric ankle fractures.

## Methods

### Specimen selection and group allocation

A total of 20 fresh-frozen cadaveric lower extremities, each allocated for single use, were included in the study after receiving ethical approval from the Institutional Review Board. All specimens were free of deformities, prior surgical procedures, history of trauma or osteoarthritis. The limbs had been amputated at the mid-tibia level. All specimens were thawed and confirmed arthroscopically to have intact joint surfaces prior to the procedure. The cadavers were randomly divided into two groups—Straight and Valgus-curved —based on the type of intramedullary nail to be used. Cadavers had a mean age of 70.1 ± 4.1 years and were balanced between groups (*p* = 0.833) in terms of age, sex (Straight nail group: 6 female; Valgus-curved nail group: 5 female, p = 1.000), and side distribution (Straight nail group: 4 right; Valgus-curved nail group: 5 right, *p* = 1.000).

### Procedural details

All procedures were performed by a single orthopedic surgeon with over 15 years of experience in foot and ankle surgery. No formal joint preparation was conducted prior to the intervention. Each specimen was stabilized in a simulated prone position before the procedure. A single Kirschner wire (K-wire) was used to maintain the foot and ankle in neutral dorsiflexion, 10 degrees of external rotation, and approximately 5 degrees of valgus alignment. The entry point was determined under fluoroscopic guidance using anteroposterior (AP), lateral, and axial Harris heel views [20]. First, on the lateral fluoroscopic image, a guidewire held next to the limb was used to establish the central longitudinal axis of the tibia, along which the first reference line was drawn. Then, on the axial heel view, a second line was drawn bisecting the calcaneal axis. The intersection of the lateral reference line and the longitudinal axis of the calcaneus was used to identify the entry point. A 3—cm central longitudinal incision was made at this intersection.

**Fig. 1:**
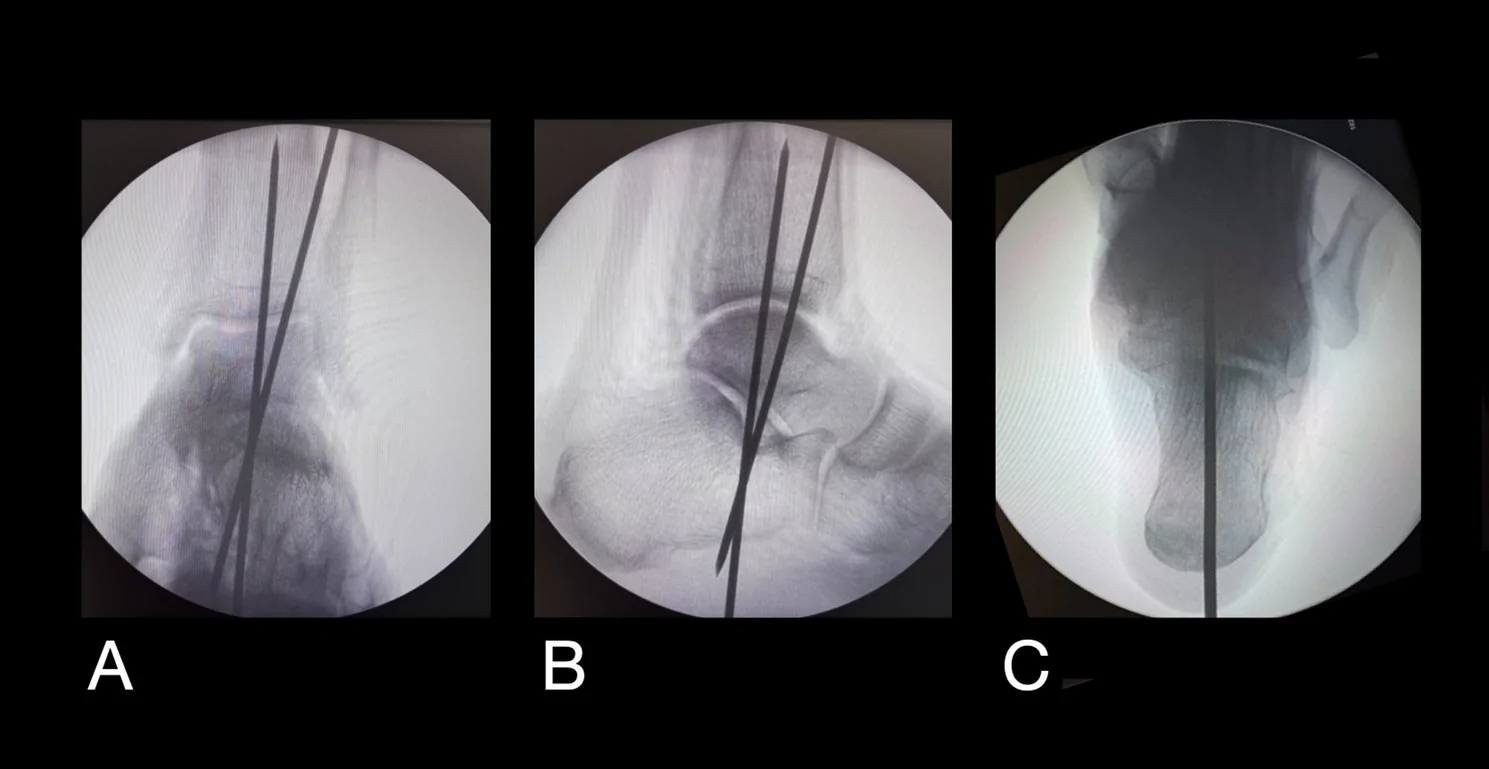
*Fluoroscopic images of straight TTC nail guidewire positioning***.** Fluoroscopic images demonstrating guidewire positioning for the straight TTC nail group. The guidewire was inserted slightly lateral to the calcaneal midline and advanced along a relatively direct calcaneus–talus–tibia trajectory to align with the tibial medullary canal. Hindfoot alignment was temporarily stabilized with an additional K-wire during guidewire insertion. **A** Anteroposterior view showing coronal plane alignment of the guidewire. **B** Lateral view confirming sagittal plane positioning. **C** Harris axial heel view illustrating the medial–lateral position of the calcaneal entry point and the coronal plane trajectory of the guidewire through the hindfoot

For the Straight nail group, the guidewire was introduced slightly lateral to the midline of the calcaneus, aligned with the tibial medullary canal, and advanced from the calcaneus to the tibia under fluoroscopic guidance. (Fig. 1) Once the adequate central position of the guidewire within the tibia was confirmed, a 12.5 mm entry reamer was used to ream the canal up to 2–3 cm proximal to the tibiotalar joint, following the manufacturer’s instructions for the Trigen Hindfoot Fusion Nail (Smith & Nephew).

**Fig. 2:**
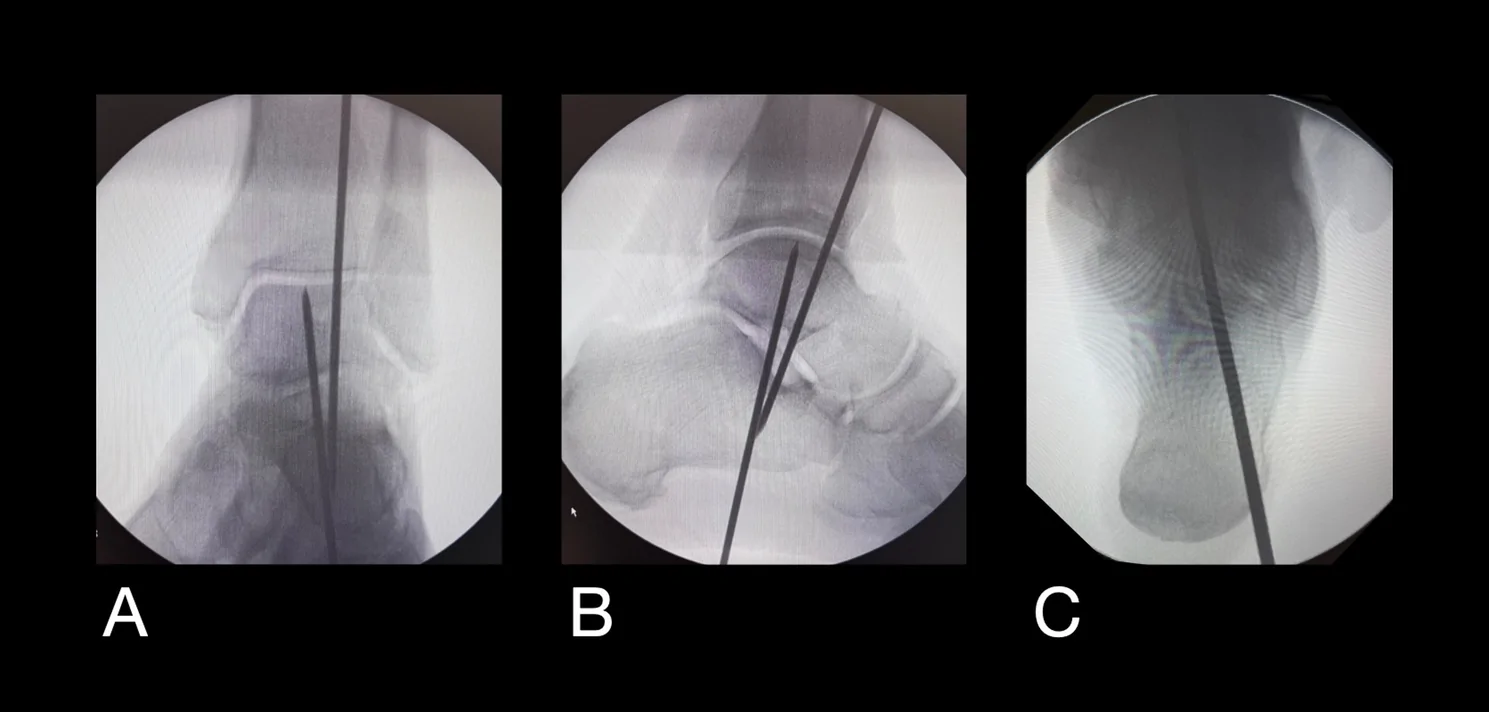
*Fluoroscopic images of valgus-curved TTC nail guidewire positioning*. Fluoroscopic images demonstrating guidewire positioning for the valgus-curved TTC nail group. The guidewire was inserted from a more lateral calcaneal entry point and directed medially toward the talus and tibial canal, consistent with the trajectory required for valgus-curved nail preparation. Hindfoot alignment was temporarily stabilized with an additional K-wire during guidewire insertion. **A** Anteroposterior view showing the valgus-oriented coronal plane trajectory. **B** Lateral view confirming sagittal plane positioning. **C** Harris axial heel view illustrating the lateralized calcaneal entry point and the coronal plane trajectory of the guidewire through the hindfoot

For the valgus-curved nail group, the plantar aponeurosis was incised parallel to the skin incision and slightly lateral to the midline to avoid interference with the entry point. The guidewire was inserted from the center of the lateral column of the calcaneus, in a more lateral position than for the straight nail, and angled medially toward the center of the talar dome. The trajectory was adjusted so that the wire exited through the central articular surface of the talus on both AP and lateral fluoroscopic views. **(**Fig. 2**)** In accordance with the manufacturer’s instructions for the Expert Hindfoot Arthrodesis Nail (DePuy Synthes), a 13 mm entry reamer was advanced through the talus until it exited proximally. Then, the positional K-wires were removed, the hindfoot was brought into inversion, and the drill tip was aligned with the center of the tibial canal on both anteroposterior and lateral views. The reamer was then advanced to create a defect in the tibial plafond. No actual TTC nail was implanted in any of the specimens after reaming in either group.

### Measurements and evaluation parameters

**Fig. 3:**
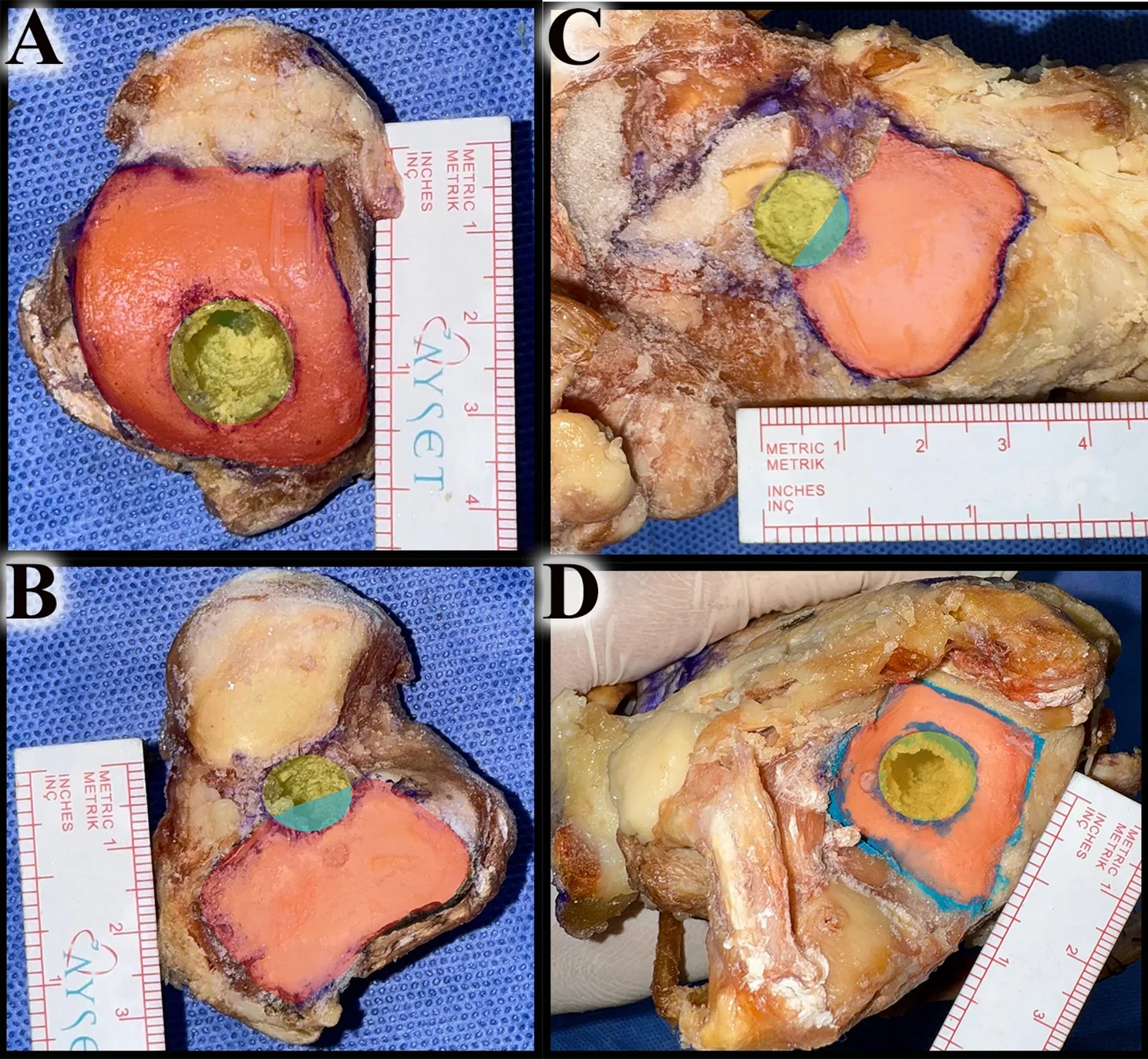
*Representative measurements of reaming-induced articular surface involvement*. Representative images showing the measurement of reaming-induced articular surface involvement after TTC nail preparation. The total joint surface area was outlined for each articular surface, and the reamed region was separately identified using calibrated digital image analysis. The red area represents the preserved articular surface where the reamer did not pass, the blue area represents the reamed portion of the articular surface, and the yellow area represents the total reamed area. Joint destruction was calculated as the ratio of the reamed articular surface area to the total joint surface area. Articular reaming was calculated as the ratio of the reamed articular surface area to the total reamed area. **A** Talar dome. **B** Posterior facet of the talus. **C** Posterior facet of the calcaneus. **D** Tibial plafond

Following the reaming procedures, all specimens were carefully dissected to expose the tibiotalar and subtalar joints. Clinical images of each joint were obtained orthogonally at a fixed distance of 12 cm from the respective articular surface [21]. The images were processed using the ImageJ software platform [22]. Before calculating the surface area, the imaging software was calibrated with a reference image of a standard ruler captured at the same angle and distance as the clinical images. Measurements were performed independently by a surgeon and an anatomist, who subsequently reached consensus on all recorded values. For each specimen, measurements detailed in Table 1 were recorded and compared, including joint destruction—defined as the ratio of reamed articular surface area to total joint surface area—and articular reaming, defined as the proportion of reamed articular surface area relative to the total reamed area, with representative measurement examples illustrated in Fig. 3**.**

**Table 1:** Measurements

Joint	Joint surface	Measurements
Subtalar joint	Posterior facet of the calcaneus (CPF)	**Total joint surface area** (cm^2^) **Remaining joint surface area after reaming** (cm^2^) **Total reamed surface area** (cm^2^) **Reamed articular surface area** (cm^2^) *Percentage of joint destruction (%):* the ratio of reamed articular surface area to total joint surface area *Articular reaming (%):* the ratio of reamed articular surface area to the total reamed surface area
	Posterior facet of the talus (TPF)	
Tibiotalar joint	Talar dome (TD)	
	Tibial plafond (TP)	

Bold text indicates the directly measured surface-area variables

### Statistical analysis

The minimum sample size required for this study was calculated using G*Power 3.1.9.7 software, based on previously reported joint destruction rates at the posterior calcaneal facet associated with different nail designs. Baker et al. reported a mean surface destruction of 10.28 ± 6.2% using a straight nail, while Lowe et al. found 4.01 ± 2.6% with a valgus-curved nail [18, 21]. Based on these values, the effect size (Cohen’s d) was 1.32, indicating that 10 specimens per group were required to detect a significant difference with 80% power at α = 0.05. Therefore, a total of 20 cadaveric specimens were randomly assigned into two groups (n = 10 each) using a simple randomization method (computer-generated random number sequence) to ensure balanced group allocation. All data analyses were conducted using IBM SPSS Statistics version 25.0 (IBM Corp., Armonk, NY, USA). The normality of data distribution was checked with the Shapiro–Wilk test. For variables that did not follow a normal distribution, the Mann–Whitney U test was used for between-group comparisons. For normally distributed variables, an independent samples t-test was applied. Continuous data are reported as mean ± standard deviation (SD), along with minimum and maximum values. A p-value of < 0.05 was considered statistically significant for all analyses.

## Results

Total joint surface area and total reamed surface area showed no significant differences between groups in CPF, TPF, TD, or TP (all *p* > 0.05). The reamed articular surface area was significantly higher in the Valgus-curved nail group for CPF (0.75 ± 0.31 vs. 0.44 ± 0.39; *p* = 0.045) and TPF (0.86 ± 0.30 vs. 0.48 ± 0.31; *p* = 0.012). No significant differences were found in TD and TP (*p* = 0.821 and 0.181, respectively). Further details regarding surface area measurements are presented in Supplementary Table 1.

The joint destruction rate in the CPF region was 12% (range 5–20%) for the Valgus-curved nail group and 6% (range 1–16%) for the Straight nail group, with a significant difference (p = 0.018). In the TPF region, the The joint destruction rate in the CPF region was 12% (range 5–20%) for the Valgus-curved nail group and 6% (range 1–16%) for the Straight nail group, with a significant difference (*p* = 0.018). group showed 15% (9–24%) and the Straight nail group 8% (2–16%), also significantly different (*p* = 0.007). No significant difference was found for joint destruction in the TD and TP regions between groups (p = 0.385 and *p* = 0.162, respectively).

The Valgus-curved nail group had higher articular reaming in both CPF and TPF regions, with 56% vs. 31% in CPF (*p* = 0.012), and 79% vs. 43% in TPF (*p* = 0.004). In TD and TP regions, articular reaming was 100% in both groups, indicating that all reamed areas were confined within the articular margins of the talar dome and tibial plafond. Additionally, reaming did not affect the anterior or medial facets of the calcaneal and talar surfaces in the subtalar joint. The results on joint destruction and articular reaming are summarized in Table 2.

**Table 2:** Assessment of articular surface destruction and articular reaming

	Joint	Total (mean±SD)	Total (min–max)	Straight (mean±SD)	Straight (min–max)	Lateral (mean±SD)	Lateral (min–max)	*p-value*
Percentage of joint destruction (%)	CPF	9.18 ± 5.49	1.0–20.03	6.37 ± 5.28	1.0–16.32	11.99 ± 4.27	5.48–20.03	**0.018**
	TPF	11.15 ± 6.25	2.29–24.01	7.6 ± 4.61	2.29–15.96	14.7 ± 5.75	9.03–24.01	**0.007**
	TD	13.04 ± 2.76	8.05–18.36	12.58 ± 2.97	8.05–18.36	13.49 ± 2.6	9.01–16.64	0.385
	TP	13.45 ± 2.41	9.31–18.42	14.07 ± 2.41	9.31–18.1	12.84 ± 2.36	10.71–18.42	0.162
Articular reaming (%)	CPF	43.59 ± 23.15	3.74–88.96	31.07 ± 23.13	3.74–63.99	56.12 ± 15.74	29.49–88.96	**0.012**
	TPF	60.76 ± 30.67	12.07–100.0	42.52 ± 25.39	12.07–100.0	79.0 ± 24.53	35.33–100.0	**0.004**

CPF, posterior facet of the calcaneus; Lateral, valgus-curved group; Straight, straight nail group; TD, talar dome; TP, tibial plafond; TPF, posterior facet of the talus.

Bold p-values indicate statistically significant between-group differences (p < 0.05)

## Discussion

This cadaveric study quantitatively compared the extent and distribution of reaming-induced articular surface destruction associated with two commonly used TTC nail designs. The principal finding was that valgus-curved nails resulted in significantly greater articular surface destruction at the posterior facets of the subtalar joint compared with straight nails, whereas no significant differences were observed at the tibial plafond or talar dome. Importantly, the absolute degree of articular surface destruction remained limited and did not exceed 15% for either nail type. Within the limitations of a cadaveric model, these results indicate that nail design affects the distribution of reaming-induced articular surface involvement, without implying clinical superiority of either construct.

TTC nails have been widely used for ankle arthrodesis, yielding favorable clinical outcomes and expanding indications over time [23, 24]. Initially advocated by Jonas et al. for use in geriatric ankle fractures, TTC nails have become a popular choice, especially in cases with significant comorbidities, poor bone quality, or fragile soft tissue, as they are often associated with shorter healing times, fewer complications, and the ability to facilitate early mobilization [1, 25, 26]. However, prior research on TTC nailing in geriatric ankle fractures often lacks detailed information regarding joint surfaces [27, 28]. As this technique is increasingly used as an alternative to conventional open reduction and internal fixation—and inherently results in permanent destruction of the tibiotalar and subtalar joint surfaces—this omission potentially creates a critical gap in the objective parameters required for surgical decision-making. In this context, our study addresses this gap by providing a quantitative and comparative analysis of articular surface damage caused by reaming for TTC nail insertion rather than attempting to define clinical superiority of one nail design over another.

The primary aim of TTC nailing in geriatric ankle fractures is to provide mechanical stabilization rather than to achieve formal arthrodesis, thereby facilitating early mobilization while minimizing perioperative medical and surgical risk. Consistent with this functional approach, an increasing number of studies have reported favorable outcomes even in the absence of formal tibiotalar or subtalar joint preparation [7, 29–32]. Reaming required for nail insertion, however, inevitably results in partial articular surface destruction, a phenomenon that has been previously documented. Baker et al. reported approximately 10% articular surface destruction at the tibiotalar and subtalar joints following reaming, whereas Lowe et al. observed about 5% involvement of the posterior subtalar facets [18, 21]. Consistent with prior cadaveric studies, reaming-associated articular surface involvement in the present series did not exceed 15% in either the tibiotalar or subtalar joints, with statistically significant differences between nail designs observed only at the posterior subtalar facets. Although residual articular cartilage has been hypothesized to permit persistent joint motion that may increase implant-related stress—particularly in younger or more active patients—the clinical significance of limited articular surface involvement in low-demand geriatric populations remains uncertain [9]. Prior cadaveric studies have suggested that comparable levels of articular surface removal are insufficient for achieving reliable fusion, and the magnitude observed in the present study aligns with these reports; however, union outcomes were not assessed [18, 21].

The posterior subtalar joint is technically distinctive due to its multiplanar orientation and considerable anatomical variability. It typically exhibits an average inclination of 42° in the sagittal plane and a medial deviation of 23° in the axial plane [33]. This complex configuration might be the reason for the reamer engaging the articular surface at a more lateral and steeper angle, particularly with valgus-curved nails inserted through the lateral column, thereby increasing the articular destruction in our study. From a methodological perspective, the use of a 0.5 mm larger entry reamer in the valgus-curved nail group, dictated by implant-specific, manufacturer-recommended instrumentation and corresponding to an approximately 4% increase in diameter, constitutes a potential confounding factor that may have contributed to the observed differences in articular destruction. However, the absence of significant between-group differences at the tibiotalar joint, in contrast to the significant differences observed at the posterior subtalar facets, suggests that reamer diameter alone is unlikely to fully account for the pattern of articular involvement. Our study shows that the valgus-curved nail group's higher articular reaming indicates that the reamer was more precisely directed at the subtalar joint surfaces, resulting in intra-articular placement of the nail. Additionally, the lack of significant differences between the two groups at the tibiotalar joint suggests that, with proper technique, consistent and predictable results can be achieved regardless of nail type.

In a cadaveric study by McGarvey et al., it was reported that a straight TTC nail penetrates the calcaneus near the junction of the sustentaculum tali and the medial calcaneal wall, resulting in poor bone purchase [12]. In another cadaveric study, Lee et al. suggested that an optimally positioned straight TTC nail would occupy approximately one-fifth of the calcaneal posterior facet [19]. Based on this, they emphasized the potential for limited medial translation of the hindfoot complex, while avoiding violation of the lateral wall, in line with previous literature [13]. Our results reported a relatively lower rate of calcaneal posterior facet destruction compared to those of Lee et al. [19]. This disparity may be attributed to methodological differences, as they employed an antegrade guidewire and calculated the affected area using a 12 mm circular outline without actual drilling. In contrast, our study simulated real surgical conditions, and the findings suggest that while controlled hindfoot medialization may be considered to optimize the placement of straight nails, this adjustment may be less necessary—or even unnecessary—when using valgus-curved nails.

Previous research suggested that positioning the entry point slightly lateral for a straight nail may improve calcaneal purchase and reduce plantar neurovascular injuries, but it could also lead to a suboptimal nail position within the tibial medullary canal [12, 20]. To prevent potential hindfoot varus from this approach, methods such as medial translation of the rearfoot complex or using short nails have been recommended [12, 34]. However, previous literature indicated that using short nails was associated with higher rates of peri-implant fractures in the geriatric population [35, 36]. In rigid coronal plane deformities, such as valgus ankle fracture malunions, the talus may not be sufficiently medialized to accommodate a standard trajectory. In such cases, alteration of the starting point and guidewire trajectory may need to be considered to obtain a more central position within both the talus and the distal tibial canal, consistent with previous reports emphasizing the noncolinear relationship between the tibial axis and hindfoot corridor [11, 14]. On the other hand, valgus-curved nails present an alternative with a reduced risk of damaging plantar neurovascular structures due to a more lateral entry point, allowing the use of long nails without the need for medialization of the hindfoot [17, 37]. Although valgus-curved nails demonstrated greater articular surface involvement in this study, this finding should be interpreted as an anatomic observation rather than evidence of clinical superiority.

Although TTC nails are often intended as a definitive treatment, nail removal may become necessary in certain cases due to implant loosening or irritation, hardware breakage, or the patient’s functional recovery expectations. In a cadaveric study by Likine et al., the authors suggested that shifting the TTC nail trajectory more anteriorly and medially could help minimize damage to the posterior facet of the subtalar joint, thereby potentially increasing the likelihood of functional recovery if implant removal is required [38]. However, they did not conduct a comparative analysis and only used a single nail design with a valgus curve. While our findings demonstrate lower articular surface involvement with straight nails, this anatomic cadaveric study does not address functional or clinical outcomes, but may help inform the design of future biomechanical experiments and hypothesis-driven clinical investigations.

This study has certain limitations that should be considered when interpreting the results. As a cadaveric study, it does not account for in vivo adjustments that often have to be made to ensure the nail is centered in the tibia with sufficient fixation in the calcaneus. In the real-world scenario, while encountering the presence of hindfoot deformity, whether using a curved nail or a straight nail, deviation from the technique guide's recommended start point may lead to trajectory changes and differences in joint destruction. A primary limitation of this study is the difference in the entry reamer diameters used between the intramedullary nail groups. In accordance with the respective manufacturer’s surgical techniques, a 13 mm reamer was utilized for the valgus-curved nail group, whereas a 12.5 mm reamer was used for the straight nail group, corresponding to a difference limited to approximately 4% in diameter. While adherence to these protocols enhances the external validity of our study by reflecting real-world clinical application, it nevertheless introduces a potential confounding variable. Additionally, sequential reaming was not performed, which may alter the contact pattern between the reamer and articular surfaces and could influence the extent of cartilage removal. The actual nails were not implanted in this study. This omission could also have implications for the percentage of joint destruction. The act of inserting the nail could further damage the joints and bone around the reamed areas. Furthermore, although joint surface destruction was measured using calibrated two-dimensional imaging, consistent with prior studies, it may not fully capture the complex three-dimensional anatomy of the hindfoot. Future studies employing three-dimensional surface scanning techniques to quantify irregular surface areas may provide a more accurate representation of reaming-induced articular destruction.

Despite these limitations, this study has several strengths. To our knowledge, it is the first cadaveric study in the literature to compare implant design in terms of reaming-induced joint surface destruction during TTC nailing. The sample size was adequate for statistical analysis, and the observed differences between groups were significant. These findings provide objective anatomic data that may inform future biomechanical investigations and hypothesis-driven clinical research.

## Conclusion

This cadaveric study demonstrated that valgus-curved TTC nails caused greater articular destruction at the posterior subtalar joint and similar amounts at the tibiotalar joint compared to straight nails; however, they did not exceed 15%. These results suggest that a significant portion of the articular surfaces remains intact in either nail design. This study highlights that TTC nail design influences the pattern and extent of articular surface involvement during reaming, but further biomechanical and outcome-focused investigations are required to clarify the clinical significance of these findings.

## Supplementary Information

Below is the link to the electronic supplementary material.

Supplementary Material 1

## Data Availability

The datasets used and/or analysed during the current study are available from the corresponding author on reasonable request.

## Abbreviations

AP: Anteroposterior
CPF: Calcaneal posterior facet
K-wire: Kirschner wire
SD: Standard deviation
TD: Talar dome
TP: Tibial plafond
TPF: Talar posterior facet
TTC: Tibiotalocalcaneal
