# Supplementary material for: Reaming-induced joint surface destruction in tibiotalocalcaneal nailing: a cadaveric comparison of straight and valgus-curved nails
Source: J Orthop Surg Res. 2026 Jul 28;21:601. doi: 10.1186/s13018-026-07136-0 (PMC13692900; doi:10.1186/s13018-026-07136-0)
Supplement: Supplementary file 1 [file 13018_2026_7136_MOESM1_ESM.docx]

**Supplementary Table 1** Measurements of Joint Surface Areas Associated with Reaming

|  | **Joint** | **Total**  (mean±SD) | **Total**  (min - max) | **Straight nail group**  (mean±SD) | **Straight nail group**  (min - max) | **Valgus-curved nail group**  (mean±SD) | **Valgus-curved nail group**  (min - max) | ***p-value*** |
| --- | --- | --- | --- | --- | --- | --- | --- | --- |
| **Total joint surface area** (cm²) | CPF | 6.44 ± 1.17 | 3.99 - 8.41 | 6.61 ± 1.34 | 3.99 - 8.41 | 6.27 ± 1.01 | 4.85 - 7.62 | 0.385 |
|  | TPF | 6.15 ± 1.18 | 4.51 - 9.07 | 6.26 ± 1.33 | 4.85 - 9.07 | 6.04 ± 1.07 | 4.51 - 7.66 | 0.970 |
|  | TD | 9.62 ± 1.34 | 7.24 - 12.61 | 9.89 ± 1.58 | 7.24 - 12.61 | 9.35 ± 1.07 | 7.34 - 10.75 | 0.571 |
|  | TP | 7.09 ± 0.94 | 5.35 - 9.38 | 7.04 ± 0.77 | 5.78 - 8.12 | 7.13 ± 1.13 | 5.35 - 9.38 | 0.970 |
| **Remaining joint surface area after reaming**  (cm²) | CPF | 5.88 ± 1.09 | 4.01 - 8.30 | 6.24 ± 1.14 | 4.59 - 8.30 | 5.52 ± 0.95 | 4.01 - 6.82 | 0.162 |
|  | TPF | 5.47 ± 1.19 | 3.58 - 8.64 | 5.78 ± 1.24 | 4.73 - 8.64 | 5.17 ± 1.12 | 3.58 - 6.96 | 0.273 |
|  | TD | 8.38 ± 1.30 | 6.17 - 11.16 | 8.66 ± 1.53 | 6.17 - 11.16 | 8.10 ± 1.02 | 6.20 - 9.16 | 0.385 |
|  | TP | 6.15 ± 0.93 | 4.36 - 8.36 | 6.06 ± 0.80 | 4.90 - 7.37 | 6.23 ± 1.09 | 4.36 - 8.36 | 0.623 |
| **Total reamed surface area** (cm²) | CPF | 1.34 ± 0.34 | 0.79 - 2.19 | 1.35 ± 0.41 | 0.79 - 2.19 | 1.32 ± 0.27 | 0.84 - 1.78 | 0.970 |
|  | TPF | 1.12 ± 0.25 | 0.71 - 1.57 | 1.12 ± 0.26 | 0.84 - 1.57 | 1.12 ± 0.26 | 0.71 - 1.45 | 0.677 |
|  | TD | 1.24 ± 0.25 | 0.80 - 1.75 | 1.23 ± 0.27 | 0.80 - 1.75 | 1.25 ± 0.25 | 0.85 - 1.70 | 0.678 |
|  | TP | 0.94 ± 0.13 | 0.66 - 1.18 | 0.98 ± 0.13 | 0.76 - 1.18 | 0.90 ± 0.12 | 0.66 - 1.03 | 0.307 |
| **Reamed articular surface area**  (cm²) | CPF | 0.59 ± 0.38 | 0.04 - 1.53 | 0.44 ± 0.39 | 0.04 - 1.27 | 0.75 ± 0.31 | 0.38 - 1.53 | **0.045** |
|  | TPF | 0.67 ± 0.36 | 0.12 - 1.45 | 0.48 ± 0.31 | 0.12 - 0.99 | 0.86 ± 0.30 | 0.51 - 1.45 | **0.012** |
|  | TD | 1.24 ± 0.25 | 0.80 - 1.75 | 1.23 ± 0.27 | 0.80 - 1.75 | 1.25 ± 0.25 | 0.85 - 1.70 | 0.678 |
|  | TP | 0.94 ± 0.13 | 0.66 - 1.18 | 0.98 ± 0.13 | 0.76 - 1.18 | 0.90 ± 0.12 | 0.66 - 1.03 | 0.307 |

Abbreviations: CPF, Posterior facet of the calcaneus; TD, Talar dome; TP, Tibial plafond; TPF, Posterior facet of the talus
